# Neuroprotective therapy after traumatic brain injury: does the window for treatment extend for months?

**DOI:** 10.1016/j.neurot.2026.e00908

**Published:** 2026-04-13

**Authors:** Cillian Lynch, Ramon Diaz-Arrastia

**Affiliations:** Department of Neurology, University of Pennsylvania Perelman School of Medicine, Philadelphia, PA 19104, USA

**Keywords:** Biomarkers, pTau, Kinase inhibitors, Neuroinflammation

Over 2% of US residents live with traumatic brain injury (TBI) related disability [1], and TBI is recognized as a risk factor for neurodegenerative dementia in late life [2]. Post-mortem histopathological examination of human brain tissues from patients dying in both the acute and chronic phases following TBI point toward a combination of neuropathological processes initiated by brain trauma but persisting for months and years that underlie these chronic neurological deficits [3]. Almost all research attempting to develop neuroprotective therapies for TBI, both in experimental models and humans, has focused on the acute period after injury [4], on the assumption that the window of opportunity to prevent secondary injury is in the order of hours, or at most a few days. This is a major limitation, as in many cases the initial injury may appear minor, and long-term disability develops in only a small fraction of cases and is evident only weeks or months later. This is particularly a problem when disability develops as a consequence of the cumulative effect of repetitive head impacts (RHIs) sustained over many years, since each impact may produce only minimal or no clinical symptoms, and disability is only evident years after RHI exposure [5].

One prominent pathogenic mechanism frequently identified immunohistochemically post-mortem after TBI is the aberrant phosphorylation of the neuronal microtubule-associated protein Tau [6]. Phosphorylated Tau (pTau) and its intracellular aggregates are features of Alzheimer's Disease (AD) [7] and AD-related dementias (ADRD) [8]. Tau phosphorylation is also prominent in acute TBI [9]. Furthermore, Tau hyperphosphorylation in perivascular glial and neurofibrillary tangles at the depths of the cerebral sulci is the pathognomonic finding of chronic traumatic encephalopathy (CTE), a pathologic diagnosis specific to individuals with a history of high level exposure to RHIs [6].

Despite extensive preclinical data providing proof of principle that neuroprotective intervention is possible, all Phase III clinical trials of therapies effective in animal models of TBI have failed to demonstrate clinical efficacy [10]. The consensus of expert panels convened by the US National Institutes of Health and the Department of Defense underscores the urgent need for biomarker-driven strategies to select participants for targeted therapeutic trials, confirm target engagement, and provide an objective measure to monitor pharmacodynamics and evaluate biologic efficacy [11,12]. Abnormal phosphorylation of neuronal Tau in the human brain is in principle identifiable by the detection of elevated levels of total Tau and pTau isoforms in circulating blood via advanced immunoassay techniques, such as the recently FDA cleared test for pTau-Thr217 as a diagnostic test for AD [13]. Tau is phosphorylated by multiple distinct protein kinases, and Tau hyperphosphorylation *in vivo* can be modulated with kinase-specific inhibitors. Thus, brain trauma-related tauopathy represents a compelling endophenotype to target in proof-of-concept preclinical therapeutic trials.

In the January 2026 issue of *Neurotherapeutics*, Melchior et al. investigate whether the pharmacological inhibition *in vivo* of a relevant kinase expressed in the brain can attenuate Tau phosphorylation at a chronic timepoint following experimental RHI [14]. The authors employ repetitive closed head impacts (once every 72–96 h, for a period of 90 days) in humanized Tau mice. SM07883, a DYRK1A kinase inhibitor being developed for the treatment of AD, was administered enterally beginning 30 days after the first impact and continued until euthanasia at 90 days following the last impact. The investigators examined the effects of RHI on motor coordination and neurobehavioral performance and characterized the degree and epitope-specificity of Tau phosphorylation in sham and injured, placebo and SM07883 treated mice via immunohistological staining and Western blot analysis.

The experimental design used by the investigators is scientifically rigorous and clinically relevant. Many studies investigating the effects of kinase inhibitors or Tau stabilizing compounds on the evolution of tauopathy in murine models of repetitive TBI have initiated drug administration following the first impact [15,16]. This approach is not directly translatable to clinical trials of subjects exposed to RHI, as patients typically come to medical attention when symptoms develop after multiple RHI exposures over several years. Longitudinal measurement of blood levels of pTau isoforms among former professional boxers show only a gradual increases in the concentration of these pre-aggregate isoforms across several years [17], suggesting that progressive Tau pathology is a chronic consequence of RHI, and that the relevant window for anti-Tau therapies is months or even years after RHI exposure. For these reasons, both the delayed administration of the inhibitory treatment, and the chronic post-injury survival phase used by Melchior et al. are particularly attractive aspects of their experimental design.

The main finding of the study is reduction in astrogliosis and microglial activation in the motor cortex, brainstem, and corpus callosum in SM07883-treated animals, compared to vehicle-treated RHI mice, assessed by immunostaining with the astrocytic marker glial fibrillary acidic protein (GFAP), and two markers of microglial activation and neuroinflammation, ionized calcium-binding adapter molecule-1 (Iba1), and CD45. There was also reduction in Tau phosphorylation detected histochemically in the brainstem as a consequence of SM07883 treatment, detected using antibodies specific for pTau-Thr231 and pTau-Ser202. Western blot analysis also confirmed reduction in pTau-Thr212 and AT8 epitopes (an antibody which recognizes paired helical filaments phosphorylated at Ser-202 and Thr-205). The investigators also report trends in improved cognitive function in SM07883-treated groups, as assessed via Barnes Maze. However, these differences did not reach statistical significance. The authors do report a significant benefit of SM07883 treatment on motor function, assessed by fall latency on the rotarod, a finding attributable in part to the reduced burden of pTau in the brainstem.

Although relatively selective for DYRK1A, SM07883 targets other kinases as well, including CLK4 and GSK3β, and DYRK1A has recognized effects on phosphorylation of other molecules that regulate innate immunity such as STAT3. Inhibition of DYRK1A in mouse models of AD reduces neuroinflammation [18], which exacerbates Tau phosphorylation in mouse models of AD and TBI [19]. [20] Although the study design by Melchior et al. is not geared toward identifying a causal mechanism for the attenuation of Tau phosphorylation on neurological sequelae following RHI, the known anti-inflammatory effects of DYRK1A inhibition [18], may in part ameliorate memory and learning deficits via rescue of hippocampal-dependent synaptic plasticity. Bergold et al. administered anti-inflammatory compounds to wild-type mice following a single closed head impact, demonstrating restoration of injury-induced decrease in hippocampal long-term potentiation (LTP) [21]. The trend in improved cognition reported by Melchior and colleagues may, thus, indicate a restorative effect of dampened neuroinflammatory response on hippocampal LTP in SM07883-treated RHI mice, in addition to any direct effects of DYRK1A on pTau accumulation in the hippocampus or brainstem.

This important study provides proof of concept that neuroprotective and neurorestorative therapies are feasible in the subacute and chronic period after RHIs, and highlights Tau phosphorylation and neuroinflammation as mechanistic endophenotypes which represent promising targets. As the investigators point out, future studies designed to determine whether SM07883 treatment reduces plasma levels of phosphorylated Tau isoforms are particularly pressing. Sensitive and specific immunoassays are now available for pTau-Thr181, pTau-Thr217, and pTau-Thr231 in a variety of platforms, including Simoa (Quaterix), MesoScale Discoveries, Elecsys (Roche), Lumipulse (Fujirebio), and others are under development. Successful clinical trials of Tau-directed therapies will almost certainly utilize these plasma biomarkers to select participants enriched in the pathology targeted by the intervention, as well as to confirm target engagement through measuring a biochemical response, which will be critical for identifying the optimal dose, timing, and treatment duration. The road to precision therapies for RHI is clear, although is likely to be long.

## Author contributions

Dr. Cillian Lynch drafted the initial version of the manuscript, which was revised and critically modified by Dr. Diaz-Arrastia.

## Declaration of competing interests

The authors declare the following financial interests/personal relationships which may be considered as potential competing interests:

Ramon Diaz-Arrastia reports financial support was provided by National Institute of Neurological Disorders and Stroke. Ramon Diaz-Arrastia reports financial support was provided by US Department of Defense. Dr. Ramon Diaz-Arrastia served as a guest editor for a special issue of Neurotherapeutics. He also served as guest reviewer for the manuscript that this commentary pertains to. He had no involvement in the peer review of this commentary. If there are other authors, they declare that they have no known competing financial interests or personal relationships that could have appeared to influence the work reported in this paper.
